# GPs' use of problem solving therapy for depression: a qualitative study of barriers to and enablers of evidence based care

**DOI:** 10.1186/1471-2296-8-24

**Published:** 2007-04-25

**Authors:** David Pierce, Jane Gunn

**Affiliations:** 1 Department of General Practice, The University of Melbourne, 200 Berkeley Street, Carlton, Victoria 3053, Australia

## Abstract

**Background:**

Depression is a major health concern, predominantly treated by general practitioners (GPs). Problem solving therapy (PST) is recognised as an effective treatment for depression that is not widely used by GPs. This research aims to explore barriers and enablers that may influence GPs use of this treatment.

**Method:**

Qualitative methodology was used including individual and focus group interviews of GPs, PST experts and consumers. Analysis was undertaken using the Theory of Planned Behaviour (TPB) as a framework.

**Results:**

A spectrum of potential influences, on GPs' use of PST emerged. Both barriers and enablers were identified. PST was perceived as being close to current practice approaches and potentially beneficial to both doctor and patient. In addition to a broadly positive attitude to PST, expressed by those with previous experience of its use, potential solutions to perceived barriers emerged. By contrast some GPs expressed fear that the use of PST would result in loss of doctor control of consultations and associated potential adverse patient outcomes. Patient expectations, which emerged as not always coinciding with GPs' perception of those expectations, were identified as a potential influence on GPs' decision concerning adoption of PST. In addition specific factors, including GP skill and confidence, consultation time constraints and technical issues related to PST were noted as potential concerns.

**Conclusion:**

This research contributes to our knowledge of the factors that may influence GPs' decisions regarding use of PST as a treatment for depression. It recognises both barriers and enablers. It suggests that for many GPs, PST is viewed in a positive light, providing encouragement to those seeking to increase the provision of PST by GPs. In identifying a number of potential barriers, along with associated options to address many of these barriers, it provides insights which may assist in the planning of GP training in PST.

## Background

Depression is a major community health concern, impacting both at the level of public health care and personal experience [1]. General practitioners (GPs) are major providers of care to those experiencing depression in the community [2]. Problem solving therapy, (PST) sometimes also called problem solving treatment or structured problem solving, is a recognised effective treatment for the management of depression in primary care [3,4]. Randomised controlled trials, conducted in general practice, suggest PST may be as effective in treating depression in this setting as antidepressant medication. It is a recommended treatment for use in general practice according to guidelines from Australia, UK and Canada [5-8]. Research has shown that most people experiencing depression prefer non-pharmacological treatments [9,10]. PST is a recognised structured psychological intervention, which aims to assist patients' awareness of links between life problems and symptoms, identification and clarification of relevant symptom related problems, goals and solutions and support patients' implementation of relevant solutions [11].

PST is regarded as easy for GPs to learn and may be provided in a small number of sessions, each limited to no more than 30 minutes. It incorporates skills that are applicable to many of the common conditions that GPs treat [12]. However, Australian research suggests that GPs' use of PST, with patients experiencing mild or moderate depression, is limited [13].

This research aimed to explore the barriers and enablers to Australian GPs' use of PST as a simple and effective management for mild to moderate major depression, in order to inform the development of a training program for GPs in PST.

## Method

Individual and focus group interviews of Australian PST experts, GPs and consumers who had experienced depression, were conducted by DP during 2005. Recruitment of PST experts was by direct invitation, based on their previous experience in both providing PST in primary care and PST education to GPs. GPs were recruited utilising the resources of a regional division of general practice* and the professional connections of the researchers. Consumers who had previously experienced depression, and had now recovered were recruited through a mental health support group. The previous experience of depression of these consumers was by self report; to participate it was not necessary for them to have heard of Problem Solving Therapy.

* (Australian divisions of general practice are government supported, local coordinating organisations for general practice.)

Individual interviews were used to collect the views of the PST experts, to allow the specific PST knowledge and experience of these participants to be gathered. Focus group interviews were chosen to collect the views of the GPs and consumers to allow the benefit of group interaction on a topic that may not be central to the thinking of the participants [14]. Three of the individual interviews were by phone due to distance constraints; all other interviews were face to face.

Focus groups and individual interviews were guided by an interview schedule. Minor adjustments were made to the interview schedules to cater for the background of group participants. Interview questions were informed by existing PST literature and recent programs in Australia to develop PST skills with GPs [15,16]. The interview schedule for GPs and PST experts explored their understanding of PST as an intervention, how they perceived it could be applied in clinical practice, barriers to its clinical use and ideas they may have to assist GPs' learning about PST. Probes were used as required to encourage discussion, particularly about the comparison of PST to other therapeutic approaches and examples of problem solving in practice. Participants were also asked to provide feedback about existing PST training material available to GPs. The interview schedule for consumers explored their understanding of PST as an intervention for mental health problems and how they perceived it could be used in clinical practice. Additional probes included consideration from a consumer (patient) perspective of time expectations, GP skill in providing PST and their views on "homework" between PST sessions.

All interviews were audio-taped and transcribed verbatim. Transcripts were read and re-read by DP to identify recurring themes. A list of themes were extracted and discussed with JG. The emerging themes were then re-read, utilising the principles of the Theory of Planned Behaviour (TPB) as a guide [17]. The TPB is a model predicting cognitive variables that influence intention to perform a behaviour; it recognises attitudes, perceived subjective norms and perceived behavioural controls as influences on behaviour. In the setting of this research it is applied to GP's decision to use, or not use, PST in consultations with patients experiencing depression.

Ethics approval for the project was provided by the University of Melbourne. All participants provided written informed consent prior to inclusion in the study.

## Results

Two focus groups of GPs were held, with six and three GPs respectively. A single focus group of six consumers was held. Four PST experts participated in individual interviews. All focus groups and interviews lasted between 60 and 90 minutes and were facilitated by DP who had previous experience facilitating both individual and focus group interviews.

Ten GPs were initially recruited; one withdrew before the focus groups were held. Six male and three female GPs participated. Their experience in general practice ranged from 2 to 31 years. Four GPs reported that for more than 25% of their adult patients, mental health was the primary issue and one GP, whose experience was as a GP and a psychiatrist, reported this proportion as more than 50%. Three of the GPs and all of the PST experts, two of whom were also GPs, had specific previous training and experience in PST. All four PST experts approached participated in the research; this group included, in addition to the two GPs already mentioned, one psychiatrist and one psychologist. Six of the seven consumers recruited participated in the consumer focus group.

Presentation of the results has been guided by the components of the Theory of Planned Behaviour.

[*Quotations are presented in italics and participants are designated as GP 01–09 for general practitioners, E 01–04 for PST experts and C 01–06 for consumers*.]

### I Attitudes to Problem Solving Therapy

Attitudes to the use of PST emerging from the interviews have been grouped as comfortable, useful, fearful and inappropriate. These are summarised in Table 1 (Summary of attitudes) below.

**Table 1:** Summary of Attitudes.

Positive attitudes	**Comfortable** Close to current practice Unlikely to cause harm Beneficial – to patient & doctor	**Useful** Pragmatic view Solutions available for PST barriers
Negative attitudes	**Fearful** Adverse patient outcome Loss of consultation control Consultation too long	**Inappropriate** Not a GP treatment Refer to other health professional

#### 1. Comfortable

Many PST experts and GP participants expressed attitudes that were broadly positive towards PST, supported by recognition that PST contains features that appeared close to GPs' current clinical practice. They were not suggesting that PST was identical to current practice, noting particularly that PST introduced a structure to familiar generic problem solving of current practice and focussed on the facilitation of patient derived solutions as distinct from the provision of advice.

*" it *[PST]*is a re-working of an old theme of a common-sense sort of step, I suppose that GPs have been using all the time, but it is less authoritarian, and it is less authoritative and it gives the patient a sort of a feeling that they are in control of their own treatment" *GP02

*"we keep saying, you are just putting into a structured form what we do." *GP06

The use of PST would therefore not require these GPs to move far from their current clinical comfort zone. This closeness may be such that some GPs may even provide PST without specifically recognising they are doing so.

*"So without even knowing it they *[GPs]*are effectively doing problem solving; they are helping the person to work through the issues themselves" *E03

The phrase "structured common sense" was used by one participant (GP) to link the structured nature of PST to the common sense, pragmatic nature of many GPs' clinical approach. Similarly, in keeping with a comfortable attitude towards PST it was seen not as a complex psychological approach, but as a relatively simple approach, consistent with this pragmatism. This view was expressed by one GP somewhat graphically:

*"so it is not brain surgery or anything complicated like that." *GP09

*"because the technique *[PST]*itself is not very hard" *E01(GP)

However one PST expert expressed a cautionary note; although PST may be very simple on the surface it may not always be properly used.

*"I think that on the surface problem solving is really simple but it is very easy to not do it properly I guess and whether a GP does it or a Psychiatrist or a Psychologist I think it is a very effective intervention." *E03

A comfortable attitude towards PST is reinforced by the perception that it is a benign approach, unlikely to harm patients, although a similar caution as that expressed above was noted about its appropriate use.

*"I can't see that the actual problem solving itself would do harm." *E03

*"You could argue that trying to solve their problems for them could do more harm than actual structured problem solving." *E02

It was also mentioned that using PST may be beneficial to the doctor as well as the patient. The GP may perceive benefits from the structure of PST, adding both boundaries to a GP's personal emotional exposure and potential for more time effective consultations.

*" problem solving workshops have actually allowed me to have some boundaries and frameworks so that I don't actually over, what is the word, over expose myself " *GP08

*"So I think that by using this technique it is a way for them to conceptualise the presentation and feel like they are doing something useful in a time efficient manner to help the person resolve the problem." *E02

Similarly the new skills PST provides the patient may limit future treatment demands and provide the GP with a more satisfying practice life.

*"If you can do this *[PST training for patient]*well once or twice with them they will learn skills that they won't hassle you so you won't have to deal with these problems as often and so frequently." *GP09

[referring to GPs who might respond to PST]*"I think those that want to get some more satisfaction out of the consultation" *E04 (GP)

This comfortable approach did not preclude frustration, associated with relinquishing control and allowing the patient to find their solution.

*"In learning the process *[using PST]*it is difficult to start with, to actually let go and allow the patient to do it and even if like you were saying if the answer is glaring you in the face they have got to come up with it to make it practical." *GP09

This comfortable attitude, whilst often being expressed by those with previous training and/or experience using PST, was also expressed by some GPs without such experience, especially as they responded to the perceived closeness of PST to current practice.

#### 2. Useful

This related, but distinct pragmatic attitude to PST, recognises both its value as a management approach for GPs, but also that its use in clinical practice is not always straightforward. This attitude was most often expressed by those with previous experience using PST in clinical practice. Perceived barriers to PST use are described and spontaneously addressed with a creative solution, suggesting the underlying attitude is one that recognises the usefulness of PST, and that this attitude is sufficiently strong to propose mechanisms to overcome perceived barriers.

Potential time difficulties may be dealt with by arranging dedicated sessions or rescheduling patients to allow effective PST use.

*"I do it once a week on a weekday afternoon where I can have a couple of patients blocked in and that for me it is blocked out time for me from an organisational point of view and it is longer consultations and therefore I am mentally prepared for it as well" *GP08

*"What is going to get you through the next week or few days or whatever?.... and then you reschedule and you make the time. You say, 'I am going to need to get you back, this is something we are going to need some time to sort out."' *GP05

Patient resistance to PST evoked the development of mechanisms to overcome such resistance. The PST approach may be personalised or tried out on a limited scale with the patient. Alternative approaches suggested include self disclosure by the GP and presenting PST as a tool, comparable to other therapeutic tools that are competently and confidently used by GPs for physical conditions.

*"I don't think you can tell that until you have tried some problem solving" *GP09

*"Can I show you a way of solving problems that I use myself?" *GP07

*"I think that problem solving is just another tool" *E01(GP)

#### 3. Fearful

##### (i) Fear of losing control of the consultation

By contrast some participants expressed a somewhat negative attitude centring on fear that the use of PST in consultations would have an adverse impact on the doctor by making consultations more difficult and on the patient by making consultations less effective. GPs with this attitude suggested that doctor control of consultations is essential. In addition they suggested that patients expected them to solve their problems, linking this perception to the doctor retaining control of the consultation.

*"I think it *[PST approach]*is putting too much onto the patient, I think the doctor has got to control the consultation." *GP01

A related approach, expressed by another GP, supported patient debating of pros and cons to solutions that had been offered by the doctor, but not generated by the patient. This approach appeared to retain firm doctor control of the consultation.

"*I think you could give them the alternatives and you can discuss the pros and cons then but once again the doctor has got to be in control of that stage." *GP06

Most of the GPs expressing fear of loss of control of the consultation did not report training or experience in PST use. By contrast those with previous experience of PST, far from being fearful of loss of control, felt PST has the potential to enhance consultation control due to the focus it may provide for both GP and patient in distressing circumstances.

*"I would hope that it *[PST]*would give GPs a somewhat greater sense of control over some of the difficult situations that are presented to them because often you have people coming in distress and they want you to help them and sometimes it is hard to know what to do and a GP can be left feeling like they are floundering" *E02

The fear that encouraging patients, especially those experiencing depression, to brainstorm solutions and make choices could both add to the patient difficulties and even be regarded as irresponsible, was expressed.

*"one of the cardinal features of depression is that you can't actually select anything, not even the colour of the shirt you are going to wear, so, and then to allow the patient, to permit the patient to select the best implementation approach to the solution, it is again almost an abrogation of the responsibility of the doctor*.*" *GP02

*"I think it could be potentially dangerous ... if they alter people's approach to a process which they intuitively arrive at over years and do quite well" *GP04

##### (ii) Fear of taking too much time

Perhaps not surprisingly the time that providing PST might require in consultations emerged as a fearful concern of some GPs. Strong single words, perhaps suggesting depth of concern, was used in response to a question about whether time issues were a concern in the context of using PST in clinical practice.

"Always."

"Absolutely."

*"Absolutely." *GPs 05,03,06

GPs who view the use of PST in consultations with fear, appeared to do so because they perceive the potential loss of consultation control, extra time required and additional patient choices as potentially resulting in an adverse patient outcome.

#### 4. Inappropriate (not the role of GPs)

A negative attitude, not to PST per se, but to its use by GPs was noted – it suggested PST, or other talking therapies if required, should be offered by a health professional other than a GP.

*"If I felt that talking therapy was then the most appropriate way to go I usually try and deflect that off onto a counsellor or something at that stage." *GP05

Consumers may reinforce this attitude, expressing the view that PST is really unsuitable for most GPs.

*"Should it really be more the domain of you know, is it too big an ask to think that just your absolutely everyday run of the mill GP might be able to adopt this?" *C04

### II Perceived social norms

Perceived normative influences on GPs' use of PST emerged from the interviews. These related to GPs' behavioural expectations; how they believed their patients expected them to act. In addition, patients discussed their expectations of GPs; how they felt their GP might or should act.

GPs' decisions to use PST may relate to their perception of patients' expectations and an implied desire to meet those patient expectations. The view introduced above, that patients expect GPs to solve their problem, was expressed by the following GP.

*"I mean they obviously come to you to with a problem and you don't have to, you don't say to them, 'You have got a problem now I am going to solve it.' That is assumed." *GP01

This perception of patient expectations is at variance with that of the following consumer who expressed a much more independent view of their relationship with their GP.

*"I no longer feel that I don't have control over my health, I have control over my health, I will ask for advice, I will accept it or reject it." *C01

The following quote from a consumer may suggest an expectation, as it might relate to GPs' use of PST, that is far removed from the above GP's perception of those expectations.

*"I guess my experience of treatment over sort of the last 20 years, would, I would, probably the GP is not the person that I would go to for treatment of depression" *C05

In addition, patients may come to the consultation with a clear expectation of the treatment they should receive; GPs may feel constrained in the treatment they can offer because of such patient expectations. This might include, (as reported from the GP's perspective), views about antidepressant medication or psychiatrist referral.

*"Some patients actually come to the consultation with fairly definite ideas about what they don't want to do.... They might come in and say I don't want to take medication I just want to see a psychiatrist." *GP04

The issue of time limitations also emerged from the patient perspective. Patient perception that time constraints currently prevent mental health work by GPs was noted, tinged with optimism for positive future change.

*"I mean at the moment they only get paid I think for 7 minutes to see a patient so you can't consult someone with mental health in 7 minutes, so I think it is valuable to use those people who have shown an interest in mental health, if it is one in five GPs around the country where there are 20,000 or something, then that is a really big base to draw off." *C04

### III Perceived behavioural limitations

Insights emerged into GPs' perception of influences that potentially might limit their provision of PST in practice. These were identified as patient factors, GP factors, PST technical factors and time constraints.

#### 1. Patient factors

Some potential patient related factors have already been noted, especially as they relate to expectations patients may bring to the consultation. More specific individual barriers were reported. Patient intelligence and a GP's response to individual patients may be significant. GPs' comments about factors that might influence their use of PST are noted below:

*"I think perhaps the intelligence of the patients." *GP06

*"So it is a kind of rapport that you are able to develop with them, yeah some people press the right buttons don't they" *GP04

Further barriers reported included concern that patients experiencing significant depression, referred to as being "cognitively stuffed" by one GP (GP07), may not be able to take the decisions required by PST.

"*It almost really makes them unable to make reasonable decisions about that *[PST]*" *GP05

Other patient factors reported that may be perceived as limiting GPs' ability to use PST included resistance to the non-traditional general practice consultation pattern associated with PST, such as the inclusion of homework and multiple treatment sessions.

#### 2. GP factors

A further range of potential barriers, related directly to GPs, were noted. Perhaps most prominent was lack of immediate agreement among GPs (and consumers) as to what PST is. Whilst the broad concept of problem solving was generally recognised by GPs, the specific term problem solving was not, with a spectrum of understanding emerging, ranging from a specific technical description, reported by those with previous training in PST, through a sense of limited familiarity with the term, to a lack of clear understanding of what the term implied. This lack of shared understanding is illustrated below

*"identification of the problem,... and coming up with alternative solutions, brainstorming, weighing up solutions, putting a plan of action in place, following up that plan of action" *E01 (GP)

*"I have, I have heard the terms but I am not sure in what sense they are being used, you mean to manage depression?" *C02

*"If problem solving was the topic I am not quite sure what you mean by that." *GP06

In addition the limited mental health training received by many GPs may limit their confidence in using PST.

[Referring to GPs' mental health skills] *"a bit unsure, unsure of their own ability a little bit threatened, feeling that they have, not have enough training. Like one doctor I was talking to yesterday and she said, 'Well I had six weeks in a psych institution and that was my training."' *E04 (GP)

#### 3. PST technical factors

Some perceived limitations related technical PST factors emerged. These included difficulty defining the problem to be addressed or attempting to solve a problem for which there is no solution;

*"Sometimes the way the punter presents things it is not entirely clear; exactly what the problem is because it is kind of presented sometimes in a jumbled way" *GP06

*"I just thought another barrier if you want to get down to the nitty gritty of it which is a very real barrier I think when you start using it *[PST]*is being able to frame what the problem is." *GP07

[referring to problems using PST] "*Trying to solve the unsolvable." *GP09

Perhaps the most prominent barrier observed by GPs was the need to change from, for some a lifetime habit of giving advice, to facilitating the patient finding a solution that they, the patient, owns. This change was recognised as being very difficult by some.

*"it is very difficult to, if you have been used to ... giving advice, especially in the organic type of frame, ... to actually stop and say, hang on no I mustn't give advice here" *E04 (GP)

*"It has got to be the patient and that *[giving advice]*is I think, that is the biggest thing that GPs stuff up" *GP07

#### 4. Time factors

Finally the perceived limitation of time constraint, which has already been reported, emerged as a perceived barrier potentially limiting GPs' decision to use PST. This limitation was seen as being related in general to the broad structure of general practice, and in particular to both mental health treatment and specific use of PST. This is reflected in the thoughts of one GP talking about his colleagues and their potential use of PST

"*I think that is a major barrier perception, a lot of GPs say to themselves, 'I can't afford to spend that much time with a patient."' *E04 (GP)

## Discussion

This research aimed to explore barriers and enablers that might influence Australian GPs' use of PST in the management of depression. It has identified a range of attitudes and perceptions, some that may act as barriers, and some that may act as enablers. This research adds to our understanding of the spectrum of relevant factors to be considered by those planning to develop PST training for GPs and those planning to implement PST as a therapeutic option in the treatment of depression by GPs.

This research has limited ability to determine the relative importance of individual factors influencing GPs' use of PST and if the observations of this research also apply in related circumstances, such as the use of PST by GPs to treat anxiety disorders. This research involved GPs with a specific interest in mental health, PST experts and consumers who have experienced depression. Although it also involved GPs without specific mental health interest, thus including the views of the wider primary health care community, the research may have included more health practitioners and consumers who are concerned about mental health issues than in these respective communities as a whole. Finally this research used a combination of focus groups and interviews; this may have impacted on the data collected. Whilst this research adds to our knowledge, further research on these issues may be appropriate.

A positive attitude towards PST as a treatment of depression was expressed by both GPs with experience of PST use and participants who had not used PST. This was reported as a positive, comfortable attitude toward PST, especially by those who recognised that it would take only a minor change from their current practice to use PST. It was seen as a useful, practical tool, likely to benefit both patient and doctor. Potential barriers to the use of PST were identified, with many participants readily offering solutions to a number of these barriers. These findings suggest that substantial enthusiasm exists towards the use of PST and this could be harnessed when implementing PST in the clinical setting.

This positive view of PST, associated with the perception that it is close to current practice, suggests the model of normalization, as an approach to facilitate the introduction of health care change, may be applicable to issues raised in this research. Normalization, in seeking to introduce a new approach, in this case the wider use of PST by GPs, aims to embed that approach as part of routine practice [18]. The limited shift, from the current practice of many GPs, required to utilize PST suggests the smooth inclusion of PST as a taken-for-granted component of practice may be relatively easy, especially if the barriers identified in this research are addressed in any such approach.

The lack of a shared understanding of terms *PST *or *problem solving *in the context of general practice treatment of depression, suggests a key starting point, for both education about PST and its use in clinical practice, should be clear information about PST; what it is and is not. Common understanding of what PST is cannot be assumed. GPs with little or no direct experience of PST are more likely to express negative attitudes towards its use. Fear of using this approach, related to perceived loss of consultation control and potential adverse outcome for patients emerged in this research as a reason for not wanting to use PST in the management of depression. Identifying GPs with such concerns is important for those promoting the use of PST in general practice. In addressing the concerns of these GPs, the positive attitude toward PST expressed by those GPs with experience in its use, suggests merit in encouraging those with fear of PST to learn more about it and try it in practice, perhaps initially using role-play as a mechanism to develop confidence in its use [19]. GPs, or patients, possessing an underlying philosophical belief that the provision of PST is inappropriate for GPs bring further challenges, including specific issues to be respectfully addressed by those training GPs in the use of PST.

Perceived social influences, reflected in GPs' view of what patients expect of them in treating depression, suggests disparity between some GPs and patients. This research, whilst alerting us to this issue, does not tell us how many patients expect their GP to solve their life problems. Given the importance of this issue for problem solving and other therapeutic approaches it is a question that should be asked in wider research.

## Conclusion

This research, which explored factors influencing GPs' use of PST in the treatment of depression, identified both barriers and enablers. In particular it suggests that for many GPs, PST is perceived in a positive light, as a comfortable approach, not far removed from current practice and that the concepts of the model of normalization may assist in the application of the issues raised in this research.

Whilst potential barriers to the use of PST by GPs emerged in the research, so did solutions to a number of these barriers. Some specific barriers identified, including GPs' fear of losing consultation control and consultation time limitations, may require specific targeted responses. This research supports the view that GP training on PST may be more effective if it is broader than the provision of technical information about PST andincludes approaches to facilitate GPs' confidence in the time applicability ofPST in general practice andshared patient-GP management approaches.

Finally, this research supports moves to increase GPs' use of PST as a treatment for depression. The enablers, barriers and potential responses to barriers it highlights may encourage increased use of PST in general practice and more focused training in PST for GPs.

## Competing interests

The authors declare that they have no competing interests.

## Authors' contributions

DP conducted the interviews reported in this research. DP and JG both contributed to project conceptual development, data analysis and manuscript preparation.

## Pre-publication history

The pre-publication history for this paper can be accessed here:


